# Baihe Dihuang Danshen Decoction Alleviates Myocardial Ischemia-Reperfusion Injury in Depression-Induced Rats by Inhibiting Ferroptosis

**DOI:** 10.2174/0113862073387437250803043736

**Published:** 2025-08-06

**Authors:** Ling Huang, Cuihua Liu, Yudan Liang, Wenquan Huang, Shizhong Zhang, Qiang Xu, Kuncheng Qiu

**Affiliations:** 1 Third-Grade Pharmacological Laboratory on Traditional Chinese Medicine, State Administration of Traditional Chinese Medicine, College of Medicine and Health Sciences, China Three Gorges University, Yichang, China;; 2 Three Gorges Public Inspection and Testing Center, Yichang, China;; 3 College of Basic Medical Sciences, Three Gorges University, Yichang, China;; 4 Yichang City Hospital of Traditional Chinese Medicine, Yichang, China

**Keywords:** Myocardial ischemia reperfusion injury, depression, baihe dihuang danshen decoction, NADPH/FSP1/CoQ10 pathway, myocardial, ferroptosis, chronic unpredictable mild stress (CUMS)

## Abstract

**Introduction:**

The comorbidity of myocardial ischemia reperfusion injury (MIRI) and depression (DEP) may worsen the prognosis of coronary heart disease surgery. Currently, research on medications and therapeutic mechanisms for MIRI combined with DEP is still insufficient. This study aims to explore the relationship between DEP and MIRI, and the therapeutic effects and mechanisms of Baihe Dihuang Danshen decoction (BDDSD) on DEP combined with MIRI.

**Methods:**

SD rats were assigned to a final experimental framework of six groups (Sham, MIRI, DEP+MIRI, BDDSD, DEP drug control, MIRI drug control). DEP was induced *via* 6-week chronic unpredictable mild stress (CUMS), with BDDSD administered during the final 2 weeks. MIRI was then induced by 30-minute coronary artery ligation and 2-hour reperfusion. DEP severity was assessed using behavioral tests (open field, elevated plus maze, sucrose preference, forced swimming). MIRI outcomes were evaluated *via* infarct size, histopathology, serum markers (LDH, IL-6, IL-1β), myocardial oxidative stress (MDA, GSH, SOD, Fe^2+^), and NADPH/FSP1/CoQ10 pathway proteins (FSP1, CoQ10, FTL, NOX2, NOX4, COX2).

**Results:**

Compared with the MIRI group, DEP significantly exacerbated MIRI, manifested by increased serum IL-6 and IL-1β levels, enlarged infarction area, and aggravated oxidative damage (elevated MDA/Fe^2+^, decreased SOD/GSH). Compared with the DEP+MIRI group,​​ BDDSD intervention relieved DEP of rats, and subsequently reduced infarction area; decreased serum LDH, IL-6, and IL-1β; lowered myocardial MDA and Fe²⁺ while increasing SOD and GSH; upregulated FSP1/CoQ10/FTL; and downregulated NOX2/NOX4/COX2 expression.

**Discussion:**

DEP can aggravate inflammation and oxidative stress, promoting cardiac ferroptosis, thereby exacerbating MIRI. Our results demonstrate that BDDSD alleviates MIRI-DEP comorbidity through a dual mechanism, mitigating depressive symptoms and inhibiting myocardial ferroptosis *via* modulation of the NADPH/FSP1/CoQ10 pathway. Although the efficacy of BDDSD is encouraging, its dose-effect relationship and long-term safety require further study.

**Conclusion:**

​​ BDDSD effectively treats DEP-MIRI comorbidity through its dual mechanism, mitigating DEP and protecting against myocardial ferroptosis. Our study not only offers a novel therapeutic strategy for patients with DEP requiring coronary heart disease surgery but also provides new targets for developing drugs to treat MIRI combined with DEP.

## 1 INTRODUCTION

Percutaneous coronary intervention and coronary artery bypass grafting are effective methods in the clinical treatment of severe coronary heart disease [1-3], but they often lead to myocardial ischemia-reperfusion injury (MIRI) [4], which impairs myocardial function. More seriously, depression (DEP) is prevalent among patients with coronary heart disease, with a prevalence rate as high as 40% [5-7], and patients with preoperative DEP have prolonged hospital stays [8] and significantly increased postoperative mortality [9, 10]. The key issue is that the combined effects of DEP and MIRI may increase the risk of adverse cardiovascular events. MIRI damages the myocardium through mechanisms such as oxidative stress [11, 12] and inflammatory responses [13, 14], while DEP exacerbates myocardial dysfunction by activating the hypothalamic-pituitary-adrenal axis, promoting platelet aggregation and so on [15-18]. This “double hit” effect can lead to more severe postoperative complications. However, research on the treatment of MIRI combined with DEP remains insufficient [19]. Therefore, exploring drugs to treat MIRI combined with DEP is of great clinical significance for improving the outcomes of coronary heart disease surgery.

However, the traditional clinical approach, such as treating DEP before surgical treatment for coronary heart disease, is not effective, because there is a complex bidirectional relationship between DEP and coronary heart disease [20]. DEP not only exacerbates the condition of patients with coronary heart disease [21-23], but the psychological and financial burden of coronary heart disease can also lead to DEP [23, 24]. Therefore, treating DEP before surgical treatment for coronary heart disease cannot avoid the occurrence of “MIRI combined with DEP”. Treating DEP and MIRI simultaneously may be a good solution. However, there are no specific drugs that can treat MIRI and DEP simultaneously. Given side effects and drug interactions, the combination of antidepressants and drugs used to treat cardiovascular diseases also has potential therapeutic risks. As people pay more attention to natural botanicals, traditional Chinese medicine (TCM) has proven to be an effective means to prevent and treat MIRI [25, 26]. Depending on the patient's specific situation, TCM is suitable for treating some complex diseases by combining different botanical drugs. It is the advantage of TCM to treat complex diseases through multi-components and multi-targets [27, 28]. Thus, TCM has great potential in the treatment of MIRI combined with DEP.

It is known that the mechanism of comorbidity between DEP and cardiovascular diseases includes inflammation, autonomic imbalance, mitochondrial dysfunction, and so on [29]. But it is unclear how DEP affects MIRI and through what mechanism. Studies have shown that inflammation is closely related to oxidative stress and ferroptosis [30-32]. Ferroptosis is one of the important pathological mechanisms of MIRI [33, 34]. DEP can cause a significant release of inflammatory cytokines (such as TNF-α, IL-1, and IL-6) in the body [35]. These inflammatory cytokines trigger a cascade of effects that lead to myocardial ferroptosis [30, 36]. Therefore, we think that DEP-induced inflammation promoting myocardial ferroptosis may be an important way for DEP to affect MIRI. When MIRI is combined with DEP, the heightened levels of inflammatory cytokines exacerbate the systemic inflammatory response, activating the NOX system. This leads to the consumption of NAD(P)H and a substantial increase in the production of reactive oxygen species (ROS) [37]. Excessive ROS induce iron metabolism disorders, triggering lipid peroxidation reactions, which ultimately lead to myocardial ferroptosis [38-40]. FSP1, by using NADPH/H as an electron source, reduces lipid free radicals under the action of CoQ10 and inhibits ferroptosis [41, 42]. Therefore, the ferroptosis signaling pathway NADPH/FSP1/CoQ10 may play an important role in the prevention and treatment of MIRI combined with DEP.

In this study, we employed BaiheDihuang-Danshen decoction (BDDSD), a novel formulation combining two classical TCM prescriptions, to treat MIRI combined with DEP in rats. BDDSD integrates the therapeutic principles of Baihe Dihuang decoction (BDD) and Danshen decoction (DSD): BDD, composed of lily bulbus and Rehmanniae radix, originates from the classical text Jin Gui Yao Lue and has been historically used to alleviate DEP [43-45]; DSD, comprising Salviae miltiorrhizae radix et rhizom, Amomi fructu, and Santali albi lignu, is clinically recognized for its anti-inflammatory and antioxidant properties in ischemic heart disease [46-48], with bioactive tanshinones notably inhibiting ferroptosis [49]. On this basis, the formulation of BDDSD strategically combines BDD and DSD to create a multi-target therapeutic approach. Our study represents the first investigation of BDDSD for MIRI-DEP comorbidity, aiming to validate its dual-pathway efficacy compared to BDD (DEP-focused) and DSD (MIRI-focused) monotherapies. To elucidate the mechanisms underlying the therapeutic effects of BDDSD on MIRI combined with DEP, we investigated the role of the NADPH/FSP1/CoQ10 pathway. Furthermore, to ensure pharmacological consistency across experimental groups,​​ we established an LC-MS-MS method to quantify seven characteristic bioactive compounds in BDDSD.

## 2 MATERIALS AND METHODS

### 2.1 Chemicals and Reagents

Malondialdehyde (MDA) and Superoxide dismutase (SOD) kits were purchased from Beijing Soleibao Technology Co., Ltd. Glutathione (GSH), Lactate dehydrogenase (LDH) and tissue Fe^2+^ kits were purchased from Nanjing Jiancheng Bioengineering Institute. IL-6, IL-1β ELISA assay kits were obtained from Shanghai Enzyme-linked Biotechnology Co., Ltd. BCA kit was supported by Beijing Applygen Technology Co., Ltd and 0.5% TTC staining solution was supported by Wuhan Saiweier Biotechnology Co., Ltd. FSP1 and β-Tubulin were purchased from Wuhan ABclonal Technology Co., Ltd. NOX2, NOX4, COX2, FTL, CoQ10, β-actin Antibodies were all obtained from Wuhan Proteintech Group, Inc. Acetonitrile, methanol, and formic acid were HPLC-grade and other reagents were of analytical grade. Reference Standards of Rehmannioside D, Tanshinol, Protocatechuic acid, Vanillic acid, regaloside A, regaloside B and Cryptotanshinone were purchased from the China National Institutes for Drug Control.

### 2.2 Animals

Male SD rats (SPF grade, 6 weeks old, 180-200 g) were purchased from the Experimental Animal Center of China Three Gorges University (License No. SCXK(E) 2022-0012). Rats were housed at 22±2°C, 60±5% humidity, under a 12-hour light-dark cycle. All procedures complied with the ARRIVE guidelines and were approved by the Ethics Committee of China Three Gorges University (No. 2024020T). Detailed measures to ensure animal welfare (*e.g.*, anesthesia and humane endpoints) are described in Section 2.4.

### 2.3 Drug Preparation and Extraction

Lily bulbus (*Lilium lancifolium* Thunb.), Rehmanniae radix (*Rehmannia glutinosa* Libosch.), Salviae miltiorrhizae radix et rhizome (*Salvia miltiorrhiza* Bge.), Amomi fructus (*Amomum villosum* Lour.) and Santali albi lignum (*Santalum album* L.) were all supported by Affiliated Renhe Hospital of China Three Gorges University, and the quality of these Herbs conforms to the provisions of the Chinese Pharmacopoeia.

The BDDSD consists of 30 g of Lily, 25 g of Rehmanniae, 30 g of Salviae miltiorrhizae, 6 g of Amomi fructus and 6 g Santali albi lignum. BDD consists of 30g of Lily and 25 g of Rehmanniae. DSD consists of 30 g of Salviae miltiorrhizae, 6 g of Salviae miltiorrhizae and 6 g of Santali albi lignum. All of the above doses are for an adult in one day. The extraction methods of BDDSD, BDD and DSD were the same. Place the herbs in a round-bottomed flask and add distilled water in a ratio of 1:6 (*w:v*). Then, reflux extraction was carried out twice, each time, for 1 hour. The filtrate was combined and concentrated to 100 mL and stored at 4°C for later use. All the decoctions were prepared every 5 days, and the dosage was converted according to the body surface area method [50]. The final intragastric dose given to the rats was calculated based on the decoction equivalent corresponding to the weight of the crude herbs: BDDSD (10.1 g/kg/day), BDD (5.7 g/kg/day), and DSD (4.4 g/kg/day).

### 2.4 MIRI Combined with DEP Rat Model and Groups

The SD rats were randomly divided into five groups by weight, including the Control group, DEP group, BDDSD group, BDD group, and DSD group (Fig. **1**). The BDD and DSD were respectively used as the DEP control drug and the MIRI control drug. There were 24 rats in the Control group and only 12 in the other groups. Besides the Control group, we used the chronic unpredictable mild stress (CUMS) method to induce DEP in other groups for 6 weeks [51, 52]. The CUMS method included fasting (24 hours), water deprivation (24 hours), overnight lighting (12 hours), wet bedding (24 hours), ice water swimming (10 minutes), and tilting the rat cage for 12 hours. One of these methods is randomly applied each day, and the same method cannot be repeated every two days. In the 7th to 8th week, the Control group was given normal saline and the BDDSD, BDD, and DSD groups were administered with the corresponding decoction, during which CUMS stimulation was maintained. At the end of the 8th week, behavioral tests were conducted to assess the degree of DEP in each group [53]. After a 24-hour rest, the Control group was divided into the Sham group and the MIRI group, with 12 rats in each group. Except for the Sham group, we performed MIRI surgery in the MIRI group, DEP group, BDDSD group, BDD group, and DSD group. The method of MIRI surgery was as follows: the rats were anesthetized with an intraperitoneal injection of 1% pentobarbital sodium (40 mg/kg), and the left anterior descending branch of the coronary artery was ligated. If the ST segment of the rat's electrocardiogram was elevated, the ligation was considered successful. The ligation was maintained for 30 minutes, followed by 2 hours of reperfusion [54, 55]. During surgery, all rats were maintained at 37 ± 0.5°C with separate heated pads. At the end of ischemia-reperfusion, rats were given an intraperitoneal injection of pentobarbital sodium (20 mg/kg) to maintain anesthesia and were euthanized by exsanguination *via* the abdominal aorta. The chest was immediately opened, and the heart tissue was rapidly excised. The collected blood was separated into serum and stored at -80°C.

### 2.5 Effects of BDDSD, BDD and DSD on DEP in Rats

#### 2.5.1 Open Field Test (OFT)

The OFT was performed in a quiet environment where each rat was gently placed in the center of an open-field arena (100 cm × 100 cm × 50 cm). The spontaneous activity of each rat, such as the duration of time spent in the central area, was observed and recorded using a video analysis system. After each test session, the open field area is thoroughly cleaned with 75% ethanol to remove any residual traces before proceeding with subsequent tests.

#### 2.5.2 Elevated Plus Maze Test (EPMT)

Firstly, the rats were acclimated to the experimental environment for 2 hours. Subsequently, each rat was placed in the central square of the maze with its head oriented towards an open arm. Concurrently, a camera monitor was activated to record the duration of time spent in the open arms, measured in seconds. In each subsequent trial, the rat was consistently positioned in the same location. Following each test, the rat's feces were removed, and the maze was cleaned with 75% ethanol to eliminate any olfactory cues that may influence the behavior in subsequent trials.

#### 2.5.3 Sucrose Preference Test (SPT)

Initially, two bottles of 1% sucrose solution were provided in each cage for a 24-hour acclimation period. A bottle of 1% sucrose solution was then replaced with distilled water, allowing the rats to acclimate for another 24 hours. After the acclimation phase, the rats were subjected to a 12-hour water deprivation period, after which the formal testing commenced. During the test, rats in each group were provided with ample amounts of both 1% sucrose solution and distilled water. After an hour, the water bottle was removed and the volume of the consumed sucrose solution and distilled water was measured. Notably, the positions of the two water bottles were switched 30 minutes after the start of the test to mitigate the effects of the rats' positional preferences [56, 57].

Sucrose preference (SP)% = sucrose consumption / [sucrose consumption + distilled water consumption] ×100.

#### 2.5.4 Forced Swimming Test (FST)

The method of FST is improved based on the literature report [58]. Rats were sequentially placed into a transparent Plexiglas cylinder (60 cm in height × 30 cm in diameter), filled to two-thirds with water at 25°C. The rats were allowed a 1-minute acclimation period within the cylinder, followed by a 10-minute observation period during which their duration of immobility was recorded.

### 2.6 Effects of BDDSD, BDD and DSD on DEP+MIRI in Rats

#### 2.6.1 Tetrazolium Chloride Staining (TTC staining)

TTC staining was used to evaluate the effect of BDDSD on myocardial infarct size [59]. MIRI surgery was performed on all rats from the MIRI, DEP, BDDSD, BDD and DSD groups. The sham group also had surgery but no coronary artery ligation. Five rats were randomly selected from each group. After MIRI surgery, the rats were killed and their hearts were fixed in an OTC embedding medium and frozen at -20°C for 30 minutes. The left ventricle was then sectioned along its longitudinal axis into five 1-2 mm transverse slices. These slices were placed in a brown bottle and covered completely with TTC staining solution, followed by a water bath at 37°C. After staining for 10-15 minutes, the staining condition was observed.

#### 2.6.2 Hematoxylin and Eosin Staining (HE staining)

HE staining was used to observe the histopathological effects of BDDSD on MIRI in rats with DEP [60]. One rat was randomly selected from each group. The rat heart was fixed with a 4% paraformaldehyde solution, dehydrated, and embedded in paraffin. Then the area near the left ventricle was cut horizontally into 4 μm sections for HE staining. The dried myocardial tissue slices were soaked in xylene and a gradient of ethanol solutions in sequence. Then, the slices were stained with hematoxylin and eosin, and dehydrated with a gradient of ethanol solutions. Finally, the slices were sealed with neutral resin and observed under a microscope.

#### 2.6.3 GSH, MDA, SOD, Fe^2+^ Levels in Rat Myocardium and IL-6, IL-1β, LDH Levels in Serum

After TTC staining and HE staining experiments, the remaining six rats in each group were subjected to abdominal aortic blood collection after MIRI surgery. The blood was allowed to clot at room temperature for 30 minutes, followed by centrifugation at 4°C at 3000 rpm for 15 minutes. The serum was collected and the levels of LDH, IL-6 and IL-1β in the serum were detected with kits. Additionally, tissue from the ligated cardiac regions of the rats was prepared into homogenates. And GSH, MDA, SOD and Fe^2+^ contents were detected with kits. IL-6 and IL-1β were primarily used to assess whether DEP affects MIRI through inflammatory factors. The MDA, SOD, LDH and GSH were used to evaluate oxidative damage of rat myocardium. The GSH and Fe^2+^ levels were used to preliminarily evaluate cardiac ferroptosis in rats.

#### 2.6.4 Detection of Ferroptosis-related Protein Expression with Western Blot

Cardiac tissue samples were collected and lysed on ice with RIPA lysis buffer. Total protein was extracted from the tissue, and protein concentration was quantified using the BCA method. Proteins of interest, such as FSP1(1:10000), NOX2(1:5000), NOX4(1:4000), CoQ10(1:1000), COX2 (1:2000) and FTL(1:6000) were separated by SDS-PAGE. The gel was then transferred to a membrane, which was incubated with primary antibodies overnight at 4°C, followed by incubation with secondary antibodies for 1 hour at room temperature. The membrane was then treated with an ECL developing solution and imaged using an imaging system. The grayscale values were analyzed using ImageJ software.

### 2.7 Content Determination of the Seven Compounds in BDDSD

We established an LC-MS-MS method for the determination of main compounds in BDDSD for quality control, including Rehmannioside D, Tanshinol, Protocatechuic acid, Vanillic acid, regaloside A, regaloside B and Cryptotanshinone. Chromatographic separation was carried out on an ACQUITY UPLC ^®^ BEH-C18 column (2.1 mm × 100 mm，1.7 μm), with acetonitrile (solution A) and -0.1% formic acid (solution B) as the mobile phase for gradient elution. The quantification of the analytes was performed by mass spectrometry with electrospray ionization (ESI) under negative and positive ion multiple reaction monitoring (MRM) mode, the drying gas temperature was 250 °C, and the sheath gas temperature was 350°C. The ion pairs we used were Rehmannioside D (709.2→362.9), Tanshinol (197.1→122.9, Protocatechuic acid (153.0→107.0), Vanillic acid (167.0→151.9), Regaloside A (399.1→163.0), Regaloside B (441.1→381.0) and Cryptotanshinone (297.1→254.0). The column temperature was 30°C and the gradient duration was 10 min at a flow rate of 0.2 mL/min as following: 5–5% A (0–1 min), 5–50% A (1–3 min), 50–95% A (3–5 min),9 5–95% A (5–7 min), 95–5% A (7-8 min), and 5–5%A (8–10 min). The injection volume was 5 µL. Before injection, the drug extract was diluted 2000 times with water and methanol as a sample solution, so that the alcohol content was 50%. We weighed seven reference standards and dissolved each in 50% methanol to a concentration of 1μg/mL. Then, an appropriate amount of each of the seven reference standard solutions was mixed to a final volume of 10 mL to prepare the mixed standard reference solution. The standard solution and sample solution were filtered with a 0.22 syringe filter before analysis.

### 2.8 Statistical Analysis

Statistical analysis was performed using GraphPad Prism 8.0.1. The data were expressed as mean ± standard deviation. Normality and homogeneity of variances were verified using the D'Agostino-Pearson and Brown-Forsythe tests, respectively. For multiple group comparisons, one-way ANOVA followed by Tukey's post hoc test was applied. All tests were two-tailed, with statistical significance defined as *P <* 0.05. Effect sizes for post hoc comparisons were calculated as Cohen’s d. Cohen’s d values were classified as large (d ≥ 0.8), medium (d ≥ 0.5), or small (d ≥ 0.2).

## 3 RESULTS

### 3.1 Effects of BDDSD on Behavior in Rats with DEP

Behavioral tests, including OFT, EPMT, SPT and FST, are often used to assess the effects of drugs on animals with DEP [61]. Since the Control group has not been further subdivided into subgroups at this point (n = 24), when conducting the tests, half of these 24 rats were randomly selected for testing to ensure that the number of rats in each group was equal (*n* = 12).

The OFT result is shown in Figs. (**2A**-**C**). Compared with the Control group, the DEP group had significantly reduced duration time in the central area (4.4±1.5 *vs.* 37.7 ± 1.9 s, *P <* 0.001, d=19.5) and total journey (373±61 *vs.* 1235±76 mm, *P <* 0.001, d=12.5), indicating that the depressed rats exhibited typical decreased exploratory behavior. Compared with the DEP group, both the BDD group and the BDDSD group significantly improved depressive behavior (both *P <* 0.05). Among them, the BDDSD group had significantly longer duration time in the central area (22.2±5.5 s) and greater total journey (1026 ± 87 mm) than the BDD group (15.2±4.0 s, 739±45 mm) (*P <* 0.01, duration time: d=1.5, total journey: d=4.1).

The EPMT result showed that the rats in the DEP group had significantly reduced duration time in the open arms (65 ± 12 *vs.* 216 ± 42 s, *P <* 0.001, d=4.9) and total journey (285 ± 78 *vs.* 1496 ± 257 mm, *P <* 0.001, d=6.4) compared with the Control group (Figs. **2D**-**F**), indicating that the depressed rats exhibited typical avoidance behavior. Compared with the DEP group, the BDDSD group had significantly increased duration time in the open arms (192 ± 27 *vs.* 65 ± 12 s, *P <* 0.01, d=6.1) and total journey (1033 ± 100 *vs.* 285 ± 78 mm, *P <* 0.01, d=8.34). The therapeutic effect of the BDDSD group was significantly better than that of the BDD group (192± 27 *vs.* 161 ± 21 s, 1033± 100 *vs.* 614 ± 125 mm) (*P <* 0.05, duration time: d=1.3, total journey: d=3.7). Although the DSD group showed a trend of improvement, there was no statistical difference. These results indicate that BDDSD can effectively enhance the rats' willingness to explore a novel environment.

Additionally, the results of the SPT and FST revealed that, compared with the Control group, the sucrose preference of the depressed rats was significantly reduced (50 ± 6% *vs.* 86 ± 5%, *P <* 0.001, d=6.5), and the immobility time in the FST was significantly increased (428 ± 28 *vs.* 324 ± 52 s, *P <* 0.01, d=2.5) (Figs. **2G**, **H**). In the SPT, compared with the DEP group, BDDSD intervention significantly increased the sucrose preference of the rats (69 ± 3% *vs.* 50 ± 6%, *P <* 0.001, d=4.0), and the BDD group also had a significant improvement (65 ± 10% *vs.* 50 ± 6%, *P <* 0.01, d=1.8), while the DSD group showed no significant change. In the FST, the BDDSD group significantly reduced the immobility time of the rats (319 ± 61 s *vs.* 428 ± 28 s, *P <* 0.01, d=2.3), and there was no significant difference compared with the Control group; the immobility time of the BDD group was shorter than that of the DEP group (338 ± 47 s *vs.* 428 ± 28 s, *P <* 0.05, d=2.8), while the DSD group showed no significant improvement.

These behavioral tests showed that BDDSD exhibited significant antidepressant effects, comparable to and even surpassing the DEP-positive control drug BDD. In contrast, the MIRI-positive drug DSD showed little improvement in DEP rats.

### 3.2 Effect of BDDSD on Myocardial Tissue Injury in rats Suffering from MIRI Combined with DEP

To evaluate the effect of BDDSD on MIRI in rats with DEP, the myocardial infarct size was measured and HE staining was performed on the injured myocardium of the rats (Figs. **3A**, **B**). Compared to the Sham group, both the MIRI and DEP+MIRI groups had myocardial infarctions (P<0.05), and the infarct size in the DEP+MIRI group was 1.5 times that of the MIRI group (P<0.05). Compared with the DEP+MIRI group, the DSD group's infarct size dropped by 41%, BDDSD by 46%, and BDD by 22.3%. BDDSD had a smaller infarct size than BDD, likely due to its antidepressant effects, enhancing the treatment of MIRI in depressed rats. Compared with the DEP + MIRI group, the myocardial infarction area in the BDD group was reduced but showed no significant difference from the MIRI group, also confirming that alleviating DEP can reduce DEP's impact on MIRI.

Compared with the Sham group, HE staining showed that myocardial cells in the MIRI and DEP+MIRI groups had disrupted myofibrils, expanded interstitial spaces, and inflammatory infiltration, with more severe damage in the DEP+MIRI group (Fig. **3C**). Compared with the DEP+MIRI group, the BDDSD and DSD groups had more orderly myocardial fibers, clearer striations, and improved inflammatory infiltration, with BDDSD performing better than DSD. The BDD group also showed some improvement over the DEP+MIRI group, likely due to BDD's antidepressant effects.

### 3.3 Effects of BDDSD on Myocardial Oxidative Injury and Inflammation in Rats Suffering from MIRI Combined with DEP

Serum LDH levels are often used to evaluate the degree of myocardial injury [62]. The serum LDH levels in DEP+MIRI and MIRI groups were significantly higher than those in the sham group (Fig. **4A**). Compared with the DEP+MIRI group, BDDSD and DSD reduced the serum LDH levels, with BDDSD demonstrating a more pronounced effect than DSD. Then we assessed the myocardial oxidative injury in myocardial tissues of rats by measuring MDA, SOD, GSH, and Fe^2+^ content (Figs. **4B**-**E**). The results revealed that myocardial oxidative injury was more severe in the MIRI+DEP group than other groups. BDDSD effectively mitigated myocardial oxidative injury in MIRI rats suffering from MIRI combined with DEP. Although DSD exhibited similar effects, its efficacy was inferior to that of BDDSD. Given that GSH and Fe^2+^ content are key mediators of ferroptosis [63, 64], the regulatory effects of BDDSD on GSH and Fe^2+^ levels suggest its potential involvement in modulating myocardial ferroptosis.

We proposed that DEP exacerbated MIRI by promoting myocardial ferroptosis by activating inflammation. Clinical studies have reported elevated serum levels of inflammatory factors in depressed patients [65-67]. Therefore, we measured serum IL-6 and IL-1β levels in each group of rats (Figs. **4F**, **G**). Compared with other groups, the serum levels of IL-6 and IL-1β were significantly elevated in the DEP+MIRI group, whereas treatment with BDDSD markedly reduced their serum concentrations. Notably, BDD exhibited regulatory effects on MDA, SOD, GSH, Fe^2+^ content, and serum IL-6 and IL-1β levels in rats, when compared with the DEP+MIRI group. These findings further substantiate our hypothesis that treating DEP may confer therapeutic benefits in managing MIRI combined with DEP.

### 3.4 Effects of BDDSD on Protein Expression Associated with NADPH/FSP1/CoQ10 Pathway

Emerging evidence suggests that the NADPH/FSP1/ CoQ10 pathway plays a pivotal role in counteracting ferroptosis [42, 68]. Our previous findings demonstrated that DEP exacerbates MIRI through inflammatory activation and oxidative stress (Fig. **4**), which are known to trigger NOX-mediated NADPH depletion and subsequent ROS overproduction. Crucially, FSP1 requires NADPH as an electron donor to reduce CoQ10, forming a key antioxidant system that prevents membrane lipid peroxidation - the hallmark of ferroptosis [69]. Therefore, we investigated the protein expression related to ferroptosis and NADPH/FSP1/CoQ10 pathway, including FSP1, CoQ10, NOX2, NOX4, FTL, and COX2.

FSP1, CoQ10, NOX2, and NOX4 are key proteins related to the FSP1 pathway. FTL reflects iron metabolism [70, 71], while COX2, a key marker of lipid peroxidation, indicates the degree of ferroptosis [72]. As shown in Fig. (5), the expression of FSP1, CoQ10, and FTL was decreased in the DEP+MIRI group (Figs. **5A**, **D**, **F**), while the expression of NOX2, NOX4 and COX2 was increased compared to the Sham group and MIRI group (Figs. **5B**, **C**, **E**). The BDD, DSD, and BDDSD groups exhibited elevated protein expression of FSP1 and CoQ10, with only the BDDSD group showing a significant increase in FTL expression compared to the DEP+MIRI group. Meanwhile, the expression of NOX2, NOX4, and COX2 was decreased in the BDD, DSD, and BDDSD groups. Compared with the BDD and DSD groups, the BDDSD group showed better regulatory effects on the expression of these related proteins. In our results, BDD also regulated the expression of these proteins. We believe that it exerted its effects on DEP+MIRI through the treatment of DEP. These results not only demonstrate that BDDSD can inhibit myocardial ferroptosis in rats by regulating the NADPH/FSP1/CoQ10 pathway, thereby alleviating MIRI, but also strongly support our hypothesis that a dual-target approach for DEP and MIRI provides better therapeutic effects for MIRI combined with DEP.

### 3.5 Content of Seven Compounds in BDDSD

Fig. (**6**) presents the chromatograms of the standard solution (Group 1) and sample solution (Group 2). Fig. (**6A**) shows the total ion chromatogram, while Fig. 6B-H displays the multiple reaction monitoring of the seven compounds: Rehmannioside D (Fig. **6B**), Tanshinol (Fig. **6C**), Protocatechuic acid (Fig. **6D**), Vanillic acid (Fig. **6E**), Regaloside A (Fig. **6F**), Regaloside B (Fig. **6G**), and Cryptotanshinone (Fig. **6H**). We measured the sample six times and recorded the peak area. The RSD values of the peak areas of Rehmannioside D, Tanshinol, Protocatechuic acid, Vanillic acid, regaloside A, regaloside B and Cryptotanshinone were 1.45%, 0.83%, 4.48%, 2.44%, 1.14%, 1.61% and 5.41%, respectively. The same sample was detected at 0 h, 2 h, 4 h, 6 h, 8h, 12 h and 24 h, the RSD values of the seven compounds were 1.59%, 2.64%, 1.82%, 0.04%, 0.01%, 0.15% and 0.23%, respectively. We used the peak area at different concentrations of the seven compounds to establish standard curves, as shown in Table **1**. Using the established standard curves, the concentration of Rehmannioside D, Tanshinol, Protocatechuic acid, Vanillic acid, regaloside A, regaloside B and Cryptotanshinone in the sample solution was 151.1±1.2, 173.0±1.6, 1.6±0.1, 8.3±0.1, 36.9±0.3, 71.1±0.6, and 4.0±0.0 μg/mL, respectively. The recovery rate was detected, as shown in Table **2**. It should be noted that the active compounds of Santali albi lignum were mainly volatile, and the proportion of Santali albi lignum in BDDSD was very low. Therefore, the active compounds in Santali albi lignum were difficult to detect, so we did not use Santali albi lignum's active compounds as quality control.

## 4 DISCUSSION

DEP combined with MIRI can raise the risk of adverse cardiovascular events after coronary heart disease surgery and may lead to more severe postoperative complications. As people pay more and more attention to the impact of DEP on cardiovascular diseases [73-75], we believe that research on MIRI combined with DEP and the search for therapeutic drugs are meaningful for the clinical surgical treatment of coronary heart disease. This study established an MIRI combined with DEP model and explored the therapeutic effects and mechanisms of BDDSD on MIRI combined with DEP, aiming to reveal the benefits of simultaneous treatment of DEP and MIRI in the process of treating "MIRI combined with DEP".

BDDSD is a novel formulation optimized based on BDD and DSD. Behavioral tests (OFT, EPMT, *etc*.) showed that BDDSD has significant antidepressant effects. Further research revealed that DEP could significantly exacerbate oxidative damage and inflammatory responses following MIRI (Fig. **4**). In a depressed state, the abnormal increase of IL-6/IL-1β activated the cardiac NOX2/4 system, causing NADPH depletion and a surge in ROS. This supports the relevant reports of the "DEP - Inflammation - Cardiovascular Injury" theory [76]. The breakthrough of this study lay in identifying ferroptosis as a bridge in this process: NADPH depletion weakened the FSP1/CoQ10 pathway's ability to suppress lipid peroxidation (as evidenced by elevated COX2, Fig. **5**), and the accumulation of free Fe²⁺ (Fig. **4E**) further worsened cardiac injury through the Fenton reaction [38, 77]. BDDSD offered a dual-regulatory treatment advantage: it alleviated DEP to reduce its impact on MIRI and inhibited cardiac ferroptosis by modulating key proteins such as FSP1. This multi-target synergistic intervention overcame the limitations of single-pathway ferroptosis inhibitors (*e.g.*, ferrostatin-1), providing a new strategy for the treatment of DEP combined with MIRI and a novel paradigm for treating complex comorbidities.

Studies show that DEP exacerbates cardiovascular diseases *via* inflammation-linked pathways, including HPA axis dysregulation, autonomic dysfunction and so on [15-18]. Our research indicates that DEP-induced inflammation promotes MIRI, but the roles of the hypothalamic-pituitary-adrenal axis and autonomic nervous system in this process require further study. In addition, we found that BDDSD can treat MIRI combined with DEP by regulating the NADPH/FSP1/CoQ10 pathway and inhibiting cardiac ferroptosis. However, the specific molecular mechanisms of this pathway remain poorly understood.

## 5 STUDY LIMITATIONS

This study explores how DEP affects MIRI and the role and mechanism of BDDSD in treating MIRI combined with DEP. Due to experimental challenges, we did not investigate the dose-efficacy relationship of BDDSD, limiting its clinical application guidance. Although we established a BDDSD quality control standard, the active components in traditional Chinese medicine are complex, and further research is needed to identify those effective for treating MIRI combined with DEP. Future research will focus on several areas. We will delve deeper into how DEP activates inflammation to promote cardiac ferroptosis and clarify the molecular mechanisms of regulating the NADPH/FSP1/CoQ10 pathway to inhibit cardiac ferroptosis. Additionally, we will screen the active components in BDDSD and conduct clinical trials on BDDSD for treating MIRI combined with DEP.

## CONCLUSION

This study shows that DEP can worsen MIRI by enhancing inflammation-driven ferroptosis. DEP-induced overexpression of IL-6/IL-1β activates the NOX system, depletes NADPH and generates excessive ROS, which promote ferroptosis and aggravate MIRI. BDDSD alleviates this comorbidity by easing DEP and inhibiting cardiac ferroptosis *via* modulating the NADPH/FSP1/CoQ10 pathway. Our study not only offers a new molecular target for MIRI combined with DEP treatment but also enhances the understanding of ferroptosis in the context of DEP and MIRI comorbidity.

## Figures and Tables

**Fig. (1):**
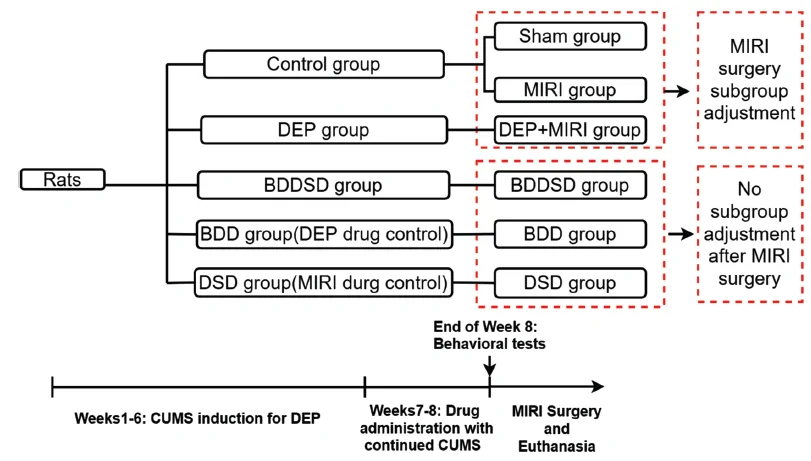
Flowchart of MIRI combined with DEP model construction and experimental grouping.

**Fig. (2):**
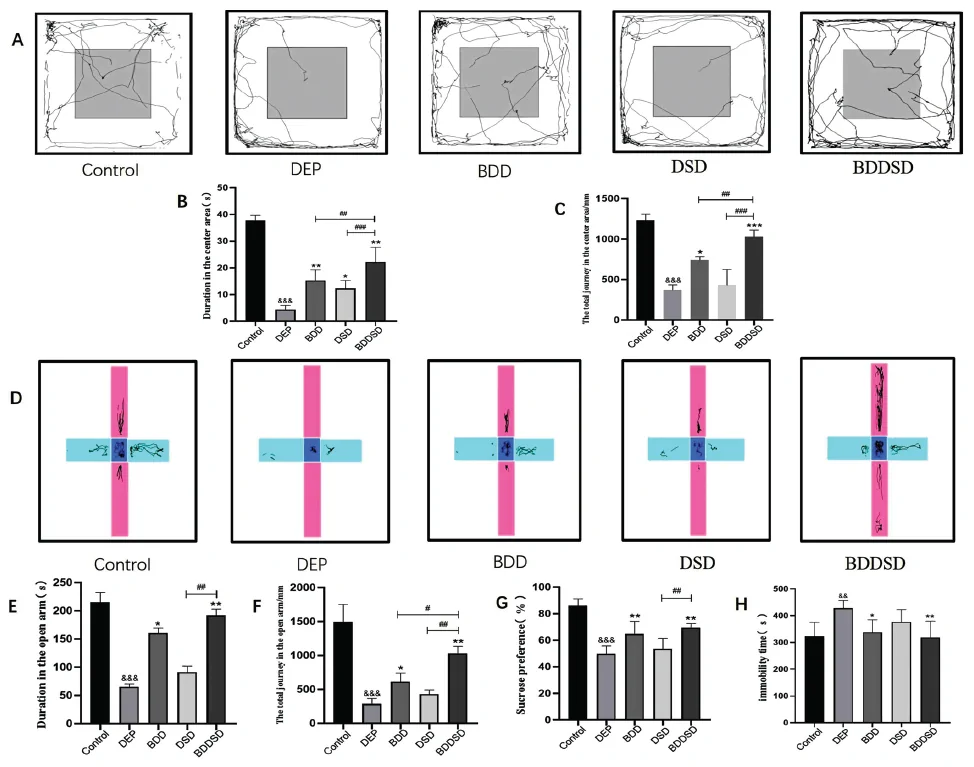
Effects of BDDSD on the behavior of rats with DEP. **(A)** Motion track in the OFT. **(B)** Duration time in the central area. **(C)** Total journey in the central area. **(D)** Motion track in the EPMT. **(E)** Duration in the open arm. **(F)** Total journey in the open arm. **(G)** Changes in sucrose preference percentage. **(H)** Immobility time. Data are expressed as mean±SD, n=12. ^&&^P<0.01, ^&&&^P<0.001 *vs.* Control group; ^*^P < 0.05, ^**^P < 0.01, ^***^P < 0.001, *vs.* DEP group.

**Fig. (3):**
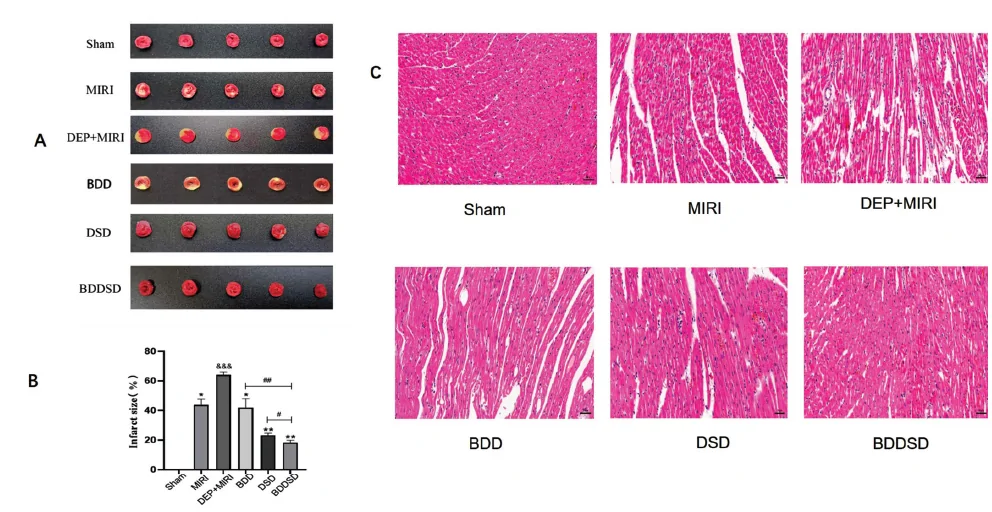
Effects of BDDSD on myocardial tissue injury in MIRI rats with DEP. **(A, B)** myocardial tissue TTC staining (*n*=5). **(C)** myocardial tissue HE staining (×200). Data are expressed as mean±SD, ^&&&^*P*<0.001 *vs.* Sham group; ^*^P < 0.05, ^**^P < 0.01, *vs.* DEP+MIRI group.

**Fig. (4):**
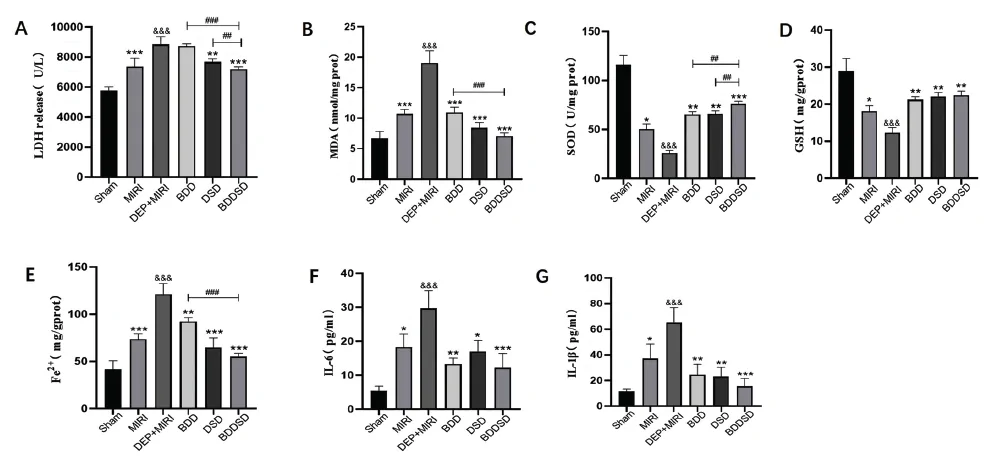
Effects of BDDSD on myocardial oxidative injury and serum inflammatory factors. **(A)** Serum LDH level. **(B-E)** Levels of MDA, SOD, GSH, and Fe^2+^ in rat myocardial tissue. **(F, G)** Levels of IL-6 and IL-1β in rat serum. Data are expressed as mean ± SD, *n* = 6, ^&&&^P<0.001 *vs.* Sham group; ^*^P <0.05, ^**^P < 0.01, ^***^P < 0.001 *vs.* DEP+MIRI group.

**Fig. (5):**
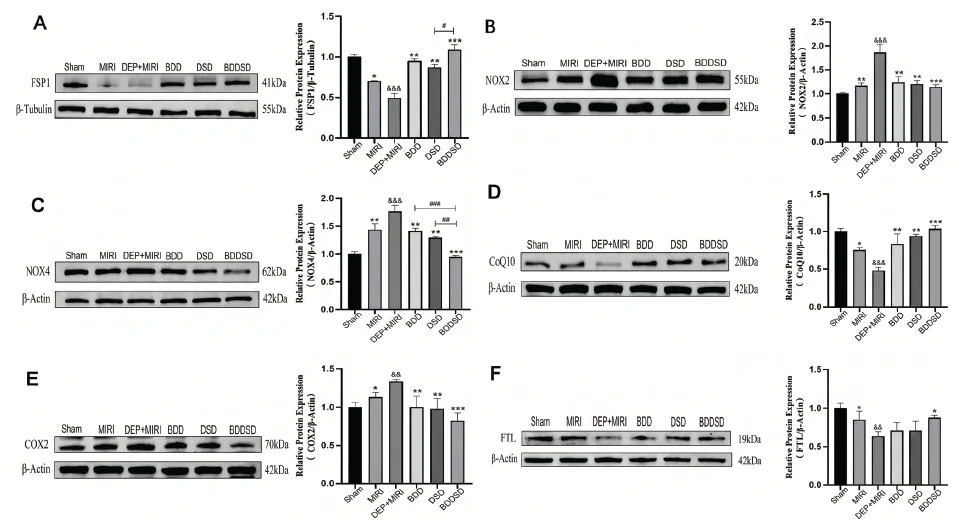
Effects of BDDSD on protein expression on ferroptosis and NADPH/FSP1/CoQ10 pathway. **(A-F)** expression levels of myocardial FSP1, NOX2, NOX4, CoQ10, COX2, and FTL. Data are expressed as mean ± SD, ^&&^*P*＜0.01, ^&&&^*P*＜0.001 *vs.* Sham group; ^*^P＜0.05, ^**^P＜0.01,^***^P＜0.001 *vs.* DEP+MIRI group;^#^*P*＜0.05,^##^*P*＜0.01,^###^*P*＜0.001 *vs.* BDDSD group.

**Fig. (6):**
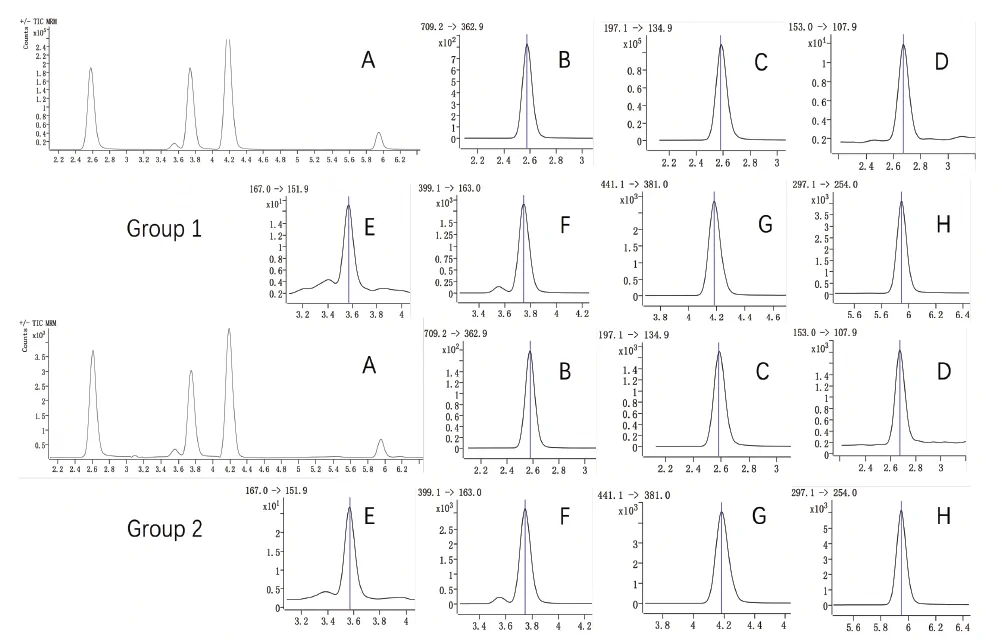
Chromatograms of standard solution (Group 1) and sample solution (Group 2). (**A**) Total ion chromatogram, (B-H) Multiple reaction monitoring of the seven compounds. (**B**) Rehmannioside D, (**C**) Tanshinol, (**D**) Protocatechuic acid, (**E**) Vanillic acid, (**F**) Regaloside A, (**G**) Regaloside B, (**H**) Cryptotanshinone.

**Table 1:** Linear equations and correlation coefficients for seven compounds.

**Reference Standard**	**Source**	**Regression equation**	**R**	**RT (min)**	**Linear Range (ng/mL)**
Rehmannioside D	Rehmanniae radix	y= 26.782x-1060.4	0.9967	2.602	67.0-511.8
Tanshinol	Salviae miltiorrhizae radix et rhizoma	y=64.841x-67.665	0.9999	2.614	20.8-200.3
Protocatechuic acid	Amomi fructus	y=59.387x-1.58	0.9993	2.700	0.2-2.0
Vanillic acid	Amomi fructus	y= 17.499x-2.021	0.9995	3.577	1.0-10.1
Regaloside A	Lily bulbus	y=465.038x-421.969	0.9997	3.750	4.5-49.8
Regaloside B	Lily bulbus	y=337.932x-121.256	0.9990	4.192	8.4-101.0
Cryptotanshinone	Salviae miltiorrhizae radix et rhizoma	y=839.620x-41.790	0.9993	5.950	0.5-4.9

**Table 2:** Concentration in sample and recovery study.

**Reference Standard**	**Concentration in Sample (ng/mL)**	**Standard Addition Concentration (ng/mL)**	**Detected Concentration (ng/mL)**	**Recovery (%)**	**Concentration in BDDSD (μg/mL)**
Rehmannioside D	75.6±0.6	75.1	75.1±0.4	99.5±1.1	151.1±1.2
Tanshinol	86.5±0.8	86.3	85.6±0.5	98.4±0.8	173.0±1.6
Protocatechuic acid	0.8±0.0	0.8	0.8±0.0	104.6±1.5	1.6±0.1
Vanillic acid	4.1±0.1	4.1	4.1±0.1	102.5±2.5	8.3±0.1
Regaloside A	18.4±0.2	18.7	18.6±0.3	104.8±3.0	36.9±0.3
Regaloside B	35.5±0.3	35.5	35.0±0.5	98.4±2.1	71.1±0.6
Cryptotanshinone	2.0±0.3	2.0	2.1±0.1	104.5±5.4	4.0±0.0

**Method:** An equal volume of standard solution with the same concentration was added to the sample solution for analysis.

**Recovery=** (Detected concentration*2-concentration in sample)/Standard addition concentration (n = 6).

Since the measured sample is diluted 2000 times, the actual concentration of each component in BDDSD is calculated.

## Data Availability

The data and supportive information are available within the article.

## LIST OF ABBREVIATIONS

BDD: Baihe Dihuang Decoction
BDDSD: Baihe Dihuang Danshen Decoction
DEP: Depression
DSD: Danshen Decoction
EPMT: Elevated Plus Maze Test
FST: Forced Swimming Test
HE: Hematoxylin and Eosin Staining
MIRI: Myocardial Ischemia-Reperfusion Injury
OFT: Open Field Test
SPT: Sucrose Preference Test
TTC: Tetrazolium Chloride Staining
